# Platelet proteome reveals features of cell death, antiviral response and viral replication in covid-19

**DOI:** 10.1038/s41420-022-01122-1

**Published:** 2022-07-16

**Authors:** Monique R. O. Trugilho, Isaclaudia G. Azevedo-Quintanilha, João S. M. Gesto, Emilly Caroline S. Moraes, Samuel C. Mandacaru, Mariana M. Campos, Douglas M. Oliveira, Suelen S. G. Dias, Viviane A. Bastos, Marlon D. M. Santos, Paulo C. Carvalho, Richard H. Valente, Eugenio D. Hottz, Fernando A. Bozza, Thiago Moreno L. Souza, Jonas Perales, Patrícia T. Bozza

**Affiliations:** 1 Center for Technological Development in Health, Oswaldo Cruz Foundation, Rio de Janeiro, Brazil; 2 Laboratory of Toxinology, Oswaldo Cruz Institute, Oswaldo Cruz Foundation, Rio de Janeiro, Brazil; 3 Laboratory of Immunopharmacology, Oswaldo Cruz Institute, Oswaldo Cruz Foundation, Rio de Janeiro, Brazil; 4 Laboratory for Structural and Computational Proteomics, Carlos Chagas Institute, Oswaldo Cruz Foundation, Curitiba, Brazil; 5 Laboratory of Immunothrombosis, Department of Biochemistry, Federal University of Juiz de Fora, Juiz de Fora, MG Brazil; 6 National Institute of Infectious Disease Evandro Chagas, Oswaldo Cruz Foundation, and D’Or Institute for Research and Education, Rio de Janeiro, Brazil; 7 National Institute for Science and Technology on Innovation on Diseases of Neglected Populations, Rio de Janeiro, Brazil

**Keywords:** Proteomics, Cell death, Mechanisms of disease

## Abstract

Coronavirus disease 2019 (COVID-19) has affected over 400 million people worldwide, leading to 6 million deaths. Among the complex symptomatology of COVID-19, hypercoagulation and thrombosis have been described to directly contribute to lethality, pointing out platelets as an important SARS-CoV-2 target. In this work, we explored the platelet proteome of COVID-19 patients through a label-free shotgun proteomics approach to identify platelet responses to infection, as well as validation experiments in a larger patient cohort. Exclusively detected proteins (EPs) and differentially expressed proteins (DEPs) were identified in the proteomic dataset and thus classified into biological processes to map pathways correlated with pathogenesis. Significant changes in the expression of proteins related to platelet activation, cell death, and antiviral response through interferon type-I were found in all patients. Since the outcome of COVID-19 varies highly among individuals, we also performed a cross-comparison of proteins found in survivors and nonsurvivors. Proteins belonging to the translation pathway were strongly highlighted in the nonsurvivor group. Moreover, the SARS-CoV-2 genome was fully sequenced in platelets from five patients, indicating viral internalization and preprocessing, with CD147 as a potential entry route. In summary, platelets play a significant role in COVID-19 pathogenesis via platelet activation, antiviral response, and disease severity.

## Introduction

Since December 2019, a new coronavirus, SARS-CoV-2 (severe acute respiratory syndrome, coronavirus 2), has been a challenge to global public health. SARS-CoV-2 has spread worldwide, affecting more than 490 million people and causing over 6 million COVID-19-related deaths since the World Health Organization declared it a pandemic [1]. Worsening of clinical outcome and severe manifestations are present in 10–20% of COVID-19 patients [2]. They have been associated with host comorbidities, especially heart disease and respiratory and metabolic syndromes [3], increasing the risk of developing complications, such as blood clotting disorders [4], platelet activation and hyperreactivity [5, 6], and thrombotic events in approximately 30% of critically ill patients [7].

Platelets are effector cells specialized in hemostasis and pathological thrombosis. In addition to functions related to aggregation [8], platelets express receptors capable of recognizing viral pathogens, as demonstrated for human immunodeficiency virus (HIV) [9], influenza virus [10], hepatitis C virus (HCV) [11], and Dengue virus (DV) [12]. These cells mediate inflammatory and immunological processes that amplify the natural response, reprogramming adjacent cells and their functions [13]. Platelet activation and platelet–leukocyte interactions are reflected in the pathophysiology of diseases, especially in inflammatory events through the release of cytokines, extrusion of neutrophil extracellular traps (NETs), and interactions with monocytes and lymphocytes [14–17]. Moreover, platelet activation and platelet-derived procoagulant extracellular vesicles (EVs) can lead to adverse hemostatic processes, that may result in the formation of thrombi, arterial ischemia, organ failure, and death [18, 19]. Although the association between thrombotic disorders in COVID-19 patients and unfavorable clinical outcomes has been established, its pathophysiological mechanisms need to be further scrutinized. Our group has investigated the role played by platelets in the pathogenesis of COVID-19, demonstrating increased platelet activation and platelet–monocyte aggregates in critically ill COVID-19 patients [5].

To deepen the knowledge about platelet biology during SARS-CoV-2 infection, this work explores the analysis of the platelet proteome. We applied a label-free shotgun proteomic approach to identify and quantify changes in platelet protein abundance in severe COVID-19 patients (survivors and nonsurvivors) compared to healthy volunteers (SARS-CoV-2-negative controls). Our results indicate that the platelet proteome undergoes expressive changes during COVID-19 infection compared to controls and between survivors and nonsurvivors, reinforcing a major role of platelets in the overall pathophysiology of COVID-19.

## Results

### COVID-19 patient cohort

**Fig. 1:**
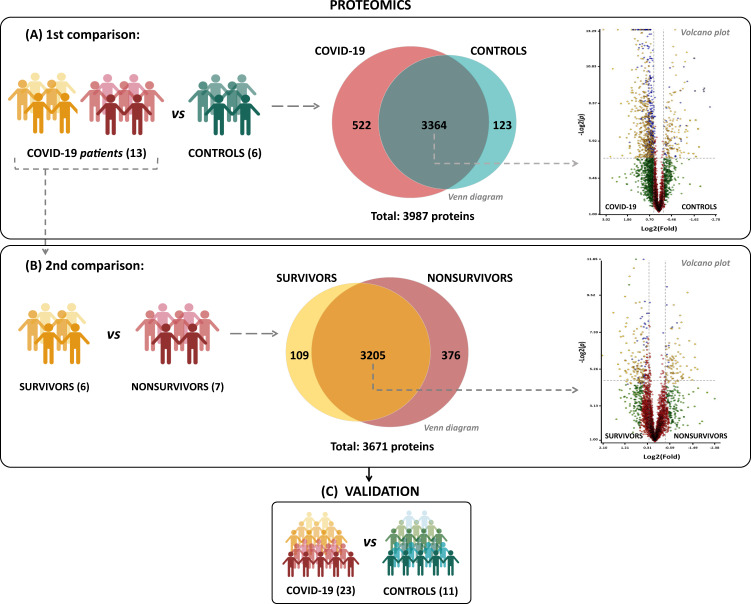
Experimental design and proteomic results. **A** Schematic representation of the experimental setup for platelet proteome screening in COVID-19 patients (COVID-19, survivor, and nonsurvivor groups) compared with healthy volunteers (controls). **B** Venn diagram representing total, unique, and shared protein identifications among the experimental groups. **C** Volcano plot of all shared proteins in the comparisons between COVID-19 patients and controls and among survivors and nonsurvivors. Each dot represents a protein mapped according to its log2 (fold change) on the *y*-axis and its −log2 (*t* test *p* value) on the *x*-axis. The red dots indicate proteins that did not satisfy the fold-change cutoff and *p* value (0.05). Green dots depict protein entries that met the fold-change cutoff but not the *p* value. Orange dots indicate proteins that satisfied both fold-change and *p* value but had low abundance, as determined by an additional stringency filter. Blue dots represent protein entries that met all statistical filters and were selected for further analysis.

The clinical profile of COVID-19 patients was matched according to Table 1. Blood samples and tracheal aspirates from 13 severe COVID-19 patients at admission to the ICU were collected, along with six age- and sex-matched healthy volunteers (control group). Patients were further classified according to the clinical outcome into two subgroups: survivors (*n* = 6) and nonsurvivors (*n* = 7). RT–PCR from tracheal aspirates or nasal swabs was then used to screen for SARS-CoV-2. All patients tested positive for SARS-CoV-2, whereas all controls had a negative result. For complementary experiments and proteomic data validation, platelets from 11 controls and 23 severe COVID-19 patients were also included (Fig. 1A and Table 1).

**Table 1:** Profile of COVID-19 patients and control subjects in proteome and validation cohorts.

Characteristics^a^	Proteome		Validation	
	Control (6)	COVID-19 (13)	Control (11)	COVID-19 (23)
Age, years	50 (36–74)	52 (39–81)	47 (26–74)	57 (33–93)
Sex, male	4 (66.66%)	6 (46.15%)	7 (63.63%)	11 (47.82%)
*Respiratory support*				
Oxygen supplementation	0 (0%)	2 (15.38%)	0 (0%)	6 (26.08%)
Mechanical ventilation	0 (0%)	11 (84.62%)	0 (0%)	17 (73.92%)
SAPS 3^b^		58 (29–84)		58.17 (29–84)
PaO_2_/FiO_2_ ratio	–	147 (0–522)	–	155 (0–522)
Vasopressor	–	7 (53.84%)	–	9 (39.13%)
Time from symptom onset to blood sample, days	–	13.5 (5–23)	–	13 (5–23)
*28-day mortality*				
Nonsurvivors	–	7 (53.84%)	–	11 (47.82%)
Survivors	–	6 (46.16%)	–	12 (52.18%)
*Comorbidities*				
Obesity	1 (16.66%)	3 (23.07%)	2 (8.69%)	4 (17.39%)
Hypertension	1 (16.66%)	6 (46.16%)	1 (4.34%)	12 (52.17%)
Diabetes	0 (0%)	4 (30.76%)	0 (0%)	9 (39.13%)
Cancer	0 (0%)	2 (15.38%)	0 (0%)	2 (8.69%)
Heart disease^c^	0 (0%)	1 (7.69%)	0 (0%)	3 (13.04%)
*Presenting symptoms*				
Cough	0 (0%)	9 (69.23%)	0 (0%)	16 (69.56%)
Fever	0 (0%)	10 (76.92%)	0 (0%)	18 (8.26%)
Dyspnea	0 (0%)	10 (76.92%)	0 (0%)	17 (73.91%)
Headache	0 (0%)	3 (23.07%)	0 (0%)	3 (13.04%)
Anosmia	0 (0%)	2 (15.38%)	0 (0%)	6 (26.08%)
*Laboratory findings on admission*				
Lymphocyte count, cells/mm^3^	–	959 (6–2584)	–	964 (6–2584)
Platelet count, ×1000/mm^3^	–	265 (131–651)	–	212 (65–651)
C reactive protein, mg/L	0.13 (0.1–0.1825)	15.16 (4.97–41.11)	0.1 (0.1–0.18)	14.41 (0.44–621.6)
Fibrinogen, mg/dL	293.1 (216.1–320.5)	581.7 (210.2–714.2)	281.5 (219.3–318.7)	545.3 (14.25–714.2)
D-dimer^d^, IU/mL < 500	5 (83.4%)	0 (0%)	10 (99.89%)	1 (4.34%)
501–3000	1 (16.66%)	5 (38.46%)	1 (0.11%)	8 (34.78%)
3001–10,000	–	5 (38.46%)	–	10 (43.47%)
>10,000	–	3 (23.07%)	–	4 (17.39%)

^a^Numerical variables are represented as the median and the interquartile range, and qualitative variables are represented as the number and the percentage.

^b^Simplified Acute Physiology Score III (scoring system for the prediction of hospital mortality).

^c^Coronary artery disease or congestive heart failure.

^d^D-dimer, specific fibrinogen degradation products (FDP) from cross-linked fibrin.

### The platelet proteome is altered during SARS-CoV-2 infection

The platelet proteome was screened through two sets of comparisons: first, the COVID-19 group was compared to the control group (Fig. 1A). Next, within the COVID-19 group, survivors and nonsurvivors were compared (Fig. 1B). In the COVID-19 vs. CONTROL comparison, we confidently identified 3987 proteins (FDR < 0.1%), of which 522 proteins were found to be exclusively detected in COVID-19 and 123 in the control, as represented in the *Venn* diagram of Fig. 1A. Exclusively detected proteins (EPs) were identified in at least two biological samples with technical triplicates in each group. Among the COVID-19 group, a set of 3671 proteins were identified, of which 109 proteins were found to be EPs to SURVIVORS and 376 to the NONSURVIVORS groups, represented in the *Venn* diagram of Fig. 1B. A list containing all information regarding peptides and protein identifications can be found in Supplemental Table 1. Proteins that were shared in both groups (intersection of *Venn* diagram) were submitted to label-free quantitation and statistical analysis with stringent criteria (*p* value < 0.05) to highlight the differentially expressed proteins (DEPs). A set of 814 proteins were found to be DEPs between COVID-19 and CONTROLS and 245 DEPs among SURVIVORS and NONSURVIVORS, represented in the volcano plots of Fig. 1A, B, respectively. The differentially abundant proteins that represented the most relevant biological processes were submitted to validation experiments in a cohort with a larger number of samples (Fig. 1C). All datasets, including fold-change and *p* values for both comparisons, are provided in Supplemental Table 2.

**Fig. 2:**
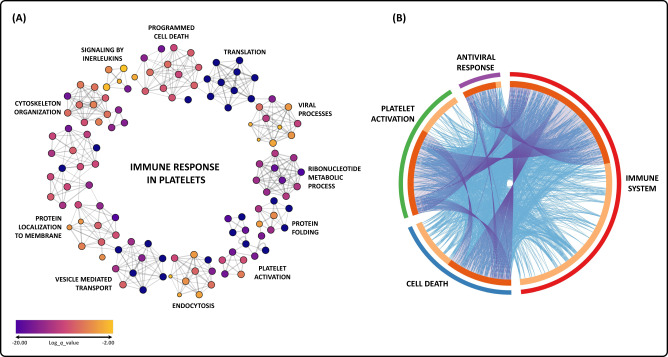
Interactome analysis and enrichment annotation of all biological processes that involve differentially abundant proteins from both comparisons in the platelet proteome of COVID-19 patients. **A** Each node represents an enrichment term statistically ranked by color. **B** Circus plot shows the overlap in differentially abundant proteins. On the outer side of the plot, the arcs represent the main biological process of each protein list. In the inner part, proteins that appear in multiple biological processes are represented in dark orange, whereas proteins unique to a biological process are depicted in light orange. Blue and purple links inside the plot indicate the degree of functional overlap among the protein lists.

Based on the results from both comparisons and focusing on EPs and DEPs, interactome analyses of enriched terms were generated to highlight the most relevant biological processes (log *P* < −3.0) (Fig. 2). The results indicate a cellular activation process that engages the immune response to SARS-CoV-2, involving several pathways, such as endocytosis, vesicle-mediated transport, cytoskeleton reorganization, protein localization to the membrane, cytokine signaling, programmed cell death, and translation (Fig. 2A and Supplemental Table 3). A protein overlapping analysis, represented by a circus plot, reinforces these results, showing that DEPs related to the immune response also directly participate in platelet activation, cell death, and antiviral response (Fig. 2B).

When comparing COVID-19 vs. controls, we found 31 EPs/DEPs assigned to platelet activation and 147 EPs/DEPs related to vesicle-mediated transport, which is the most statistically significant biological process (Supplemental Fig. 1 and Supplemental Table 3). Consistently, our recent study showed that the majority of plasma EVs from patients with severe COVID-19 are platelet-derived [19]. Regarding platelet activation, Von Willebrand factor (vWF) and prothrombin (F2) were downregulated in the COVID-19 group. The receptor for thromboxane A2 (TBXA2R), a potent stimulator of platelet aggregation, was detected as EP in the COVID-19 and survivor groups. In addition, platelet-derived growth factor C (PDGFC) and receptor alpha (PDGFRA) were found to be more abundant in the COVID-19 group, with PDGFRA displaying such a trend only in the survivor subgroup (Supplemental Table 2a and e). Our group recently demonstrated increased platelet activation in the tracheal aspirate from severe patients by measuring plasma levels of TXB_2_ (a metabolite from platelet TXA_2_) and PDGF (a platelet alpha-granule protein), suggesting that products from platelet activation and secretion may gain access to the airways, correlating with the severity of COVID-19 syndrome [5].

### The platelet proteome from COVID-19 patients presents a molecular phenotype of cell death

**Fig. 3:**
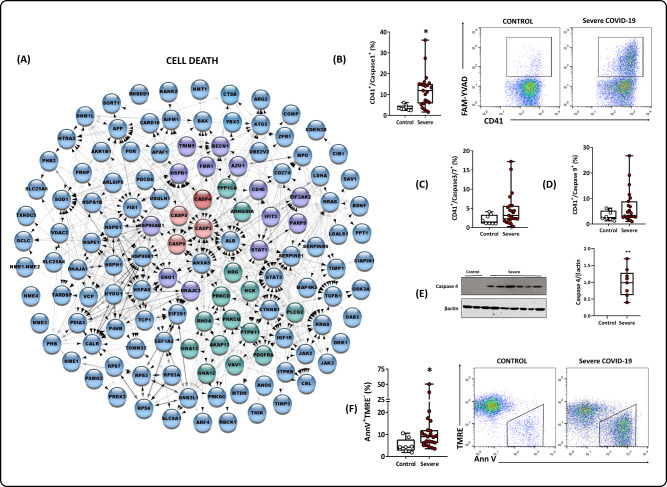
Cell death regulation in the platelet proteome. **A** PPI related to programmed cell death and the apoptotic process. Colors represent biological processes. Proteins in blue are exclusive to programmed cell death and apoptotic processes. Proteins in green and purple are shared with PPI platelet activation and PPI response to type I interferon, respectively. In red, caspases 3 (DEP) and caspases 4, 5 and 9 (EPs) were upregulated in the COVID-19 group. **B** Caspase-1 activation in platelets isolated from healthy volunteers (controls) and patients with severe COVID-19. **C** Caspase 3/7 activation in platelets isolated from controls and patients with severe COVID-19. **D** Caspase 9 activation in platelets isolated from controls and patients with severe COVID-19 (percentage of positive cells by flow cytometry). **E** Western blot analysis of caspase 4 and β-actin expression in platelets isolated from three control subjects and 11 patients with severe COVID-19; and graph demonstrating band densitometry. **F** Cell death was evidenced by phosphatidylserine exposure (Annexin V positive) and collapse of the mitochondrial membrane potential (TMRE negative) in platelets isolated from control and patients with severe COVID-19 (percentage of cells by flow cytometry). Representative dot plots are shown in (**B**) and (**F**). Boxes indicate the median and interquartile ranges, and whiskers indicate minimal and maximal values in each group. *p < 0.05 compared to control with severe COVID-19 (unpaired *t* test).

An in vitro study reported that SARS-CoV-2 internalization leads platelets to programmed cell death, and EVs release [20]. The proteomic comparison between COVID-19 and controls extended these findings, highlighting DEPs related to vesicle-mediated transport, as mentioned above (Supplemental Fig. 1), and 129 DEPs related to cell death (Fig. 3A). Specifically, in the cell death pathway, caspases 3, 4, 5, and 9 were detected as markers for apoptosis and pyroptosis. We used flow cytometry and western blotting as an orthogonal validation of our findings. We observed a significant increase in activated caspase 1 (Fig. 3B) and a milder activation of the effector complex formed by caspases 3 and 7 (Fig. 3C). We also observed a positive trend toward the activation of caspase 9 (Fig. 3D). The abundance of caspase 4 (Fig. 3E) was also increased; notably, caspase 4 is a critical cysteine proteinase in the intrinsic pathway and the endoplasmic reticulum stress (ER stress) and pyroptosis responses. A pro-cell death stage was observed by proteomic DEP ANXA5, confirmed through an increase in tagged annexin V (Fig. 3F) and a decrease in the uptake of tetramethylrhodamine (TMRE) (Fig. 3F).

### Antiviral response through type-I interferon is significantly augmented in platelets from COVID-19 patients

**Fig. 4:**
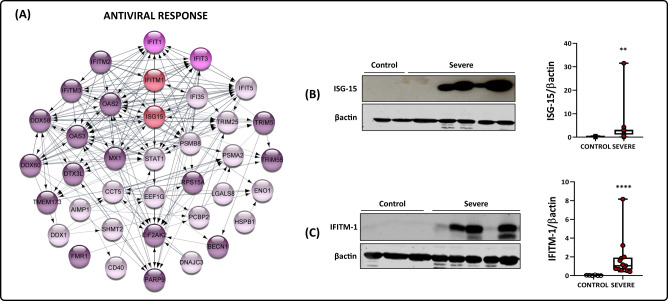
Antiviral response in the platelet proteome. **A** PPI is related to the response through type-I interferon. Each color represents a different EP/DEP classification: purple EPs represent PPIs described in the COVID-19 group. The EPs IFIT1 and 3, EPs from the COVID-19 group, and a DEP exclusive to the survivor group subset are represented in pink. The DEPs from the COVID-19 group are displayed in light pink. Last, the validated proteins ISG-15 and IFITM-1, both EPs from the COVID-19 group, are represented in red. All DEPs were shown to be upregulated. **B** Western blot analysis of ISG-15 and β-actin in platelets isolated from three healthy volunteers (controls) and nine patients with severe COVID-19. **C** Western blot analysis of IFITM-1 and β-actin in platelets isolated from three controls and nine patients with severe COVID-19. The densitometric analyses of each band are represented in a graph. The boxes indicate the median and interquartile ranges, and whiskers indicate minimal and maximal values in each group. *p < 0.05 between controls and severe COVID-19 patients (unpaired *t* test).

Previous transcriptomics studies reported the antiviral response pathway as differential platelet gene expression from patients with COVID-19 [6, 20]. In this work, we detected 44 EPs/DEPs from the interferon-stimulated gene (ISG) family, such as IFI35, IFIT1, IFITM1, IFITM2, and IFITM3 (Fig. 4A). Some were also directly related to effectors of cell death processes, such as STAT1, STAT3, ANXA5, CASP3, CASP5, CASP4, CASP9, BAX, and APAF1 (Fig. 3A). Interferon signaling is classically described as the first line of defense against viral infections, reducing disease progression and allowing the body to assemble an effective response [21]. Type-I interferons induce the expression of ISGs through binding to the IFNα receptor complex (IFNAR) [22].

Among the ISGs found to be induced by SARS-CoV-2, the interferon-induced 35 kDa protein (IFI35) is involved in the innate antiviral response [23]. In contrast, the family of interferon-induced proteins with tetratricopeptide repeats (IFITs), particularly IFIT1 and IFIT3, are correlated with translation blockage of viral proteins by binding to eukaryotic initiation factor 3 (eIF3) or through mRNA sequestration [24]. Higher levels of induced transmembrane protein IFITM3 mRNA were observed in platelets during influenza A/H1N1 and dengue [25]. In COVID-19, the IFITM3 gene was recently found to be upregulated in platelets [6] and epithelial cells [26]. Polymorphism of IFTM3 was also correlated with higher disease severity [27]. Western blot analyses showed significant expression of ISG-15 and IFITM-1 (Fig. 4B, C), in line with proteomic results showing these EPs from the COVID-19 group. The data suggest the importance of platelets as effectors in the host’s antiviral response to infection, shedding light on yet another role of platelet function that has been insufficiently addressed.

### Platelets internalize the virus and express proteins related to translation, mainly in samples from nonsurviving patients

**Fig. 5:**
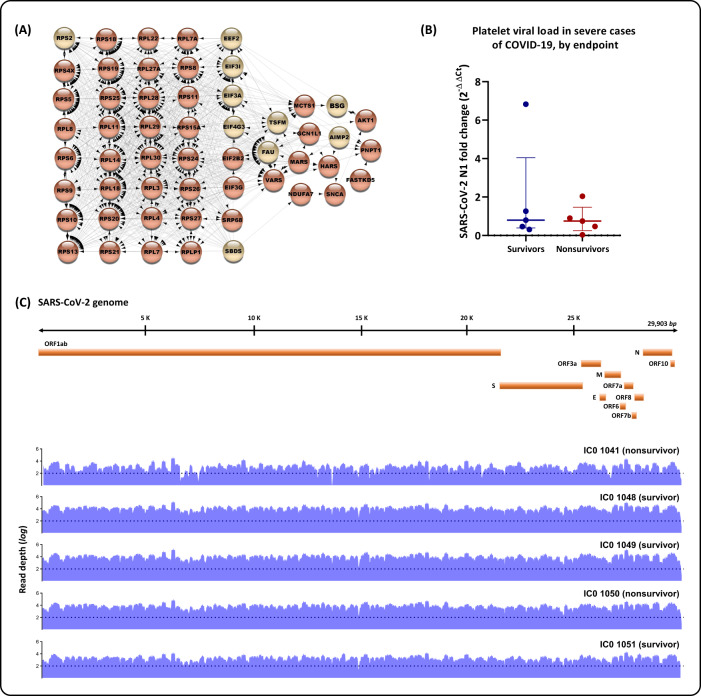
Platelet infection by SARS-CoV-2. **A** PPI is related to mRNA translation and structural constituents of ribosomes. Circles in dark orange represent EPs from nonsurvivors; circles in light orange represent DEPs more abundant in the overall COVID-19 group. **B** Platelet viral loads in severe cases of COVID-19, by endpoint: survivors or nonsurvivors. No significant differences were found between endpoints (Mann–Whitney *U* = 11, *P* = 0.8413, ns). **C** SARS-CoV-2 genome coverage graphs of platelet samples. Platelet RNA was submitted to SARS-CoV-2 target sequencing to screen for viral genome presence, whether total or partial. A scheme of the Wuhan-Hu-1 genomic isolate [GenBank: NC_045512.2], spanning all structural and nonstructural components, is represented with coverage graphs (blue) for each sample [GenBank: OL984052; OL984053; OL986402; OL984057; OL984061]. Dotted lines mark read depths equal to 100.

The mRNA processing and translation pathways were highlighted when comparing survivors and nonsurvivors (Fig. 2). Several ribosomal proteins were detected as nonsurvivor EPs, along with eukaryotic translation factors, detected as upregulated DEPs (EIF3A, EIF3I, and EIF4G3) or EPs found in the nonsurvivor group (EEF2 and EIF2B2) (Fig. 5A and Supplemental Table 2b and f). Particularly, these eukaryotic translation factors are related to major events in protein translation and are described as essential factors in virus replication [28]. It was previously reported that platelets can sustain the translation and RNA synthesis of the dengue virus, but mature viral particles were not assembled [29]. More recent studies have reported the internalization of SARS-CoV-2 in patients’ megakaryocytes and platelets; nevertheless, genomic analysis was unable to unequivocally demonstrate a full-length virus genome [20, 26]. Interestingly, we identified the BSG protein, also named CD147, as a DEP in COVID-19 patient samples (Fig. 5A and Supplemental Table 2A). This protein has been reported as an unconventional receptor for platelet activation and virus internalization [30, 31].

To further investigate whether platelets could be infected by the virus, we quantified the viral RNA levels through RT–PCR in platelets from ten randomly chosen patients and found consistent molecular detection in these samples from survivors and nonsurvivors (Fig. 5B). A more sensitive protocol was used when compared to other studies [32], leading to the sequencing of the complete SARS-CoV-2 genome, with >98% coverage and >100× depth, in platelet samples from five representative COVID-19 patients (Fig. 5C and Supplemental Table 4). The coverage and depth criteria employed in this study to generate consensus genome sequencing are consistent with the best next-generation sequencing practices to implicate a virus in a sample.

Based on this evidence, we reanalyzed the raw proteomics data with a *de novo* sequence approach to search for viral peptides that could have been translated. However, no viral peptides/proteins were unequivocally identified (FDR < 1%). Nevertheless, our data indicate that viral RNA is present in the platelets of survivors and nonsurvivors, but only in the latter would translationally related proteins be engaged. To analyze the effect of SARS-CoV-2 entry into platelets, we infected cells in vitro with SARS-CoV-2. We observed no generation of infective virus within 24 or 48 h compared to Vero E6 or Calu-3 cells. These cells are considered efficient in generating infectious progeny, as measured by plaque-forming units (Supplemental Fig. 2). Additionally, incubation of platelets from severe COVID-19 patients positive for SARS-CoV-2 genetic material through RT–PCR with Vero E6 cells did not result in cytopathic effects (data not shown), indicating that platelets were not carrying infectious viral particles.

## Discussion

The physiopathology of COVID-19 is multilayered and involves several cellular targets [33]. To gather new insights into the molecular mechanisms associated with platelets during COVID-19 infection, we performed an in-depth proteomic analysis of platelets from patients infected with SARS-CoV-2 and healthy volunteers. Since platelets are anucleated cells, transcriptome and proteomics techniques have been used as tools to evaluate platelet activation in several diseases. Through the proteomics technique in sepsis, it has already been observed that the increase in the expression of integrin aIIbB3 in platelets of patients and mice correlated with mortality [34]. Additionally, in dengue, when analyzing the proteomics of platelets of patients, our group showed that there is an increase in platelet activation and inflammatory responses and in other functions that were not previously linked to platelets, such as processing and presentation of antigens, proteasome activity and histone expression [12].

The identified and quantified proteome was then submitted to two comparative analyses—controls vs. COVID-19 and survivors vs. nonsurvivors—to screen for differentially abundant proteins that could be correlated with disease progression. Proteomic analyses of platelets from controls vs. platelets from COVID-19 patients highlighted proteins from several pathways, but three biological processes stood out: “platelet activation”, “cell death” and “type I interferon”. Platelet activation has been considered a hallmark of COVID-19 since patients often have mild thrombocytopenia and appear to have increased platelet consumption along with a corresponding increase in platelet production [35]. Additionally, previous studies have shown that platelets isolated from critically ill COVID-19 patients can activate neutrophils and induce the release of neutrophil extracellular traps (NETs) [6] and the induction of several cytokines and chemokines, pointing to a direct contribution of platelets to the plasma cytokine storm [36–38]. Identifying several procoagulant proteins reinforces the findings previously reported in the literature, demonstrating that platelets from COVID-19 patients are hyperreactive and that abnormal platelet reactivity may also lead to hypercoagulability and augmented proinflammatory activities.

Cell death mechanisms in COVID-19 have been reported in prior studies involving clinical samples and in vitro experiments with different cell lines, including platelets [20, 39]. In this context, we identified several caspases as differentially expressed in our proteomic dataset, indicating active caspase-driven cell death processes in platelets. Caspase-1 and caspase-4 were further validated in functional experiments. These proteins are considered proinflammatory caspases and promoters of pyroptosis, a specific cell death process part of the host’s response to viral infections [40]. Viral PAMPs and DAMPs (pathogen/danger-associated molecular patterns) lead to inflammasome assembly, which in turn elicits the processing of pro-caspase-1. Cleaved caspase-1 promotes the processing of gasdermin-D, the effector molecule of pyroptosis, and the release of proinflammatory cytokines [41]. Cytokine processing is also an augmented pathway in platelets from COVID-19 patients in our proteomic cross-comparisons. Caspase-1 is also very important in the release of cytokines such as IL-1b and may be involved in this process. We recently showed that platelets from critically ill COVID-19 patients induce IL-1β secretion in monocytes [42]. However, platelets from critically ill COVID-19 patients do not secrete higher levels of IL-1β than control platelets when cultured overnight. Platelets infected with SARS-CoV-2 in vitro also did not secrete IL-1β [42]. Nevertheless, Zaid et al. showed that platelets from COVID-19 patients secrete higher levels of IL-1β under subthreshold thrombin stimulation than control platelets [37].

In line with these findings, Koupenova et al*.* [20] demonstrated that in the platelet transcriptome of patients with COVID-19, there is an upregulation of pathways related to cell death, specifically apoptosis and necroptosis. In addition, they observed by immunofluorescence the colocalization of SARS-CoV-2 with phospho-MLKL (mixed lineage kinase domain-like pseudokinase) and caspase 3 in nonpermeabilized platelets in vitro and COVID-19 platelets [20]. Additionally, a recent study with rhesus monkeys indicated an upregulation in caspase-1 in peripheral blood cells from day 2 postinfection [43]. In conclusion, the data suggest that pyroptosis is the primary cell death pathway in platelets from severe COVID-19 patients.

The proteomic data also point to platelet involvement in an essential cross-talk mechanism through cytokine processing and signaling pathways, namely, the interferon response pathway (IFN). Once infected, cells undergo viral recognition through pattern recognition receptors that trigger interferon-stimulated (ISG) gene expression, which activates signal transduction pathways that lead to the production of more interferons and procytokines. Our dataset highlighted interferon-stimulated gene-15 (ISG-15) as differentially abundant in critically ill patients. The expression of ISG-15 is strongly associated with an antiviral response, reducing viral replication, minimizing the externalization of virions, and acting as a chemotactic cytokine for neutrophils [44]. Recently, a multiomics analysis in a model of severe COVID-19 also highlighted the importance of ISG-15 in the overall antiviral response, particularly in the lungs [45]. The same phenomenon has already been observed when infecting the megakaryocyte cell line MEG-01 with dengue virus and observed an increase in the expression of ISG-15, which is important in the antiviral response [46]. The recruitment of other interferon-induced protein families [tetratricopeptide repeat proteins (IFITs) and IFN-induced transmembrane proteins (IFITMs)] was also highlighted by our data. Briefly, these protein families are involved in translation initiation, virus replication, double-stranded RNA signaling, cell migration, and proliferation [24] and significantly influence the disease progression and outcome of several viral infections [47]. IFITM3 was upregulated in platelets isolated from patients during clinical infections of influenza and dengue virus; due to some modification, there is a lower expression in platelets, and there is an increase in disease severity and mortality in patients. Infection of human MKs with DENV selectively increased type I and IFITM3 interferons, and overexpression of IFITM3 was sufficient to prevent infection [25]. Previous work has shown increased expression of IFITM3 in platelets from COVID-19 patients [6]; our dataset includes the differential abundance of ISG15 and IFITM1, reinforcing the importance of platelets in antiviral response.

The clinical progression of COVID-19 can be highly variable among individuals, ranging from mild, asymptomatic infections to severe respiratory distress. Accordingly, the molecular background of each patient plays a significant role in determining the disease outcome [48]. In this context, we classified the severe COVID-19 patients according to their clinical outcomes (survivors/nonsurvivors) and compared their proteomic datasets. Notably, the DEPs/EPs were, in a significant majority, related to mRNA translation pathways. These proteins are components of the small ribosomal 40S subunit and are required for rRNA processing and 18S rRNA maturation [49]. Coronavirus mRNA relies on cap-dependent translation to produce viral proteins enhanced in *trans* by the SARS-CoV N protein [50]. The overrepresentation of the proteins related to cap recognition by the 40S ribosomal subunit might suggest an initial step of viral protein translation in platelets. Similar behavior was also observed for Dengue virus and influenza, which have been described to promote platelet activation, thus allowing infection and RNA synthesis [10, 51]. Furthermore, it has been demonstrated that nonstructural protein 1 from SARS-CoV-2 can recruit the 40S ribosomal unit [52], and a progenitor megakaryocyte cell line (MEG-1) was shown to express the N protein 48 h post-incubation with SARS-CoV-2 [53]. Additionally, recovery of full-length viral RNA from platelets suggests SARS-CoV-2 internalization. This event might have triggered translational mechanisms in platelets for nonsurvivors. Consistently, the SARS-CoV-2 positive- and negative-sense RNA have been detected in platelets from patients in association with fatal outcomes [54]. Other studies have partially documented the internalization of SARS-CoV-2 in platelets [6, 25, 37, 55]. SARS-CoV-2 does not actively replicate in platelets, and the presence of viral RNA could influence the activation of intracellular PAMPs and downstream signaling events of activation and death [20, 30], as well as the transfer of the SARS-CoV-2 genome to the target cells [54]. Although we cannot say that translation phenomena are occurring in platelets, it is known that in addition to chemokines stored in granules and immune receptors, these platelet components include RNA molecules and spliceosomes from megakaryocytes [56]. Thus, platelets contain RNA molecules and activation-dependent posttranscriptional mechanisms to process intronic RNA, allowing the synthesis of immunoregulatory proteins such as interleukin (IL)-1β and tissue factor (TF) and antimicrobial peptides such as β-defensins, among others [56]. In addition, transcriptomic and transplastomic studies have shown evidence of platelet translation [34, 57–59]. The ability of platelets to translate viral RNA has also been reported [29, 51, 60], which strengthens the possibility that the phenomena observed in this work are occurring in platelets.

However, the route of SARS-CoV-2 entry into platelets is still unclear. Previous reports showed no evidence of ACE2 mRNA expression in platelets either from COVID-19 patients or from cultured megakaryocytes from healthy donors [6], suggesting that the internalization of SARS-CoV-2 in megakaryocytes and platelets might occur through alternative pathways. A screening experiment based on the receptor subdomain 1 of the SARS-CoV-2 spike protein (RBD-SD1) demonstrated a strong tropism of RBD-SD1 to bone marrow cells, even though there is a substantially low level of ACE-2 expression in the tissue [61]. RNA-Seq experiments have indicated that the receptors CD147, KREMEN1, and NRP-1 are potential routes for internalization [53]. Of them, the CD147 transmembrane protein was further shown to lead to SARS-CoV-2 endocytosis in ACE2-deficient T cells [62], and SARS-CoV-2 or recombinant S protein activates platelets through CD147 [30]. The identification of CD147 as a DEP in platelets from COVID-19 patients supports its role as an alternative route for SARS-COV-2 internalization.

Altogether, our findings support an important role for platelets in the host’s thromboinflammation and antiviral response during COVID-19. The internalization of SARS-CoV-2 in platelets, evidenced by the complete genome of SARS-CoV-2 and a higher expression level of proteins related to viral mRNA translation, suggests quasi-functional cellular machinery for mounting viral particles. Despite being unable to promote replication and further infection, these cellular products could trigger disease progression, thus representing potential markers for COVID-19 severity. Finally, our data indicate profound molecular alterations, possibly involved in both protective and pathological immune responses, which may be related to COVID-19 clinical outcomes.

## Methods

### Human subjects

Peripheral venous blood samples were obtained from 23 patients with severe COVID-19 admitted within 72 h from ICU admission in three reference centers (Instituto Estadual do Cérebro Paulo Niemeyer, Hospital Copa Star, and Leblon Campaign Hospital, all in Rio de Janeiro, Brazil), whose characteristics are presented in Table 1. Severe COVID-19 was defined as those critically ill patients, presenting viral pneumonia on computed tomography scan and requiring oxygen supplementation through either a nonrebreather mask or mechanical ventilation [63]. Clinical information from all severe COVID-19 patients was collected using a standardized form—ISARIC/WHO Clinical Characterization Protocol for Severe Emerging Infections (CCP-BR) [63]. Blood samples were collected within 72 h of ICU admission. All patients had a confirmed diagnosis of SARS-CoV-2 through RT–PCR of nasal swabs and/or tracheal aspirates. Peripheral vein blood was also collected from 13 SARS-CoV-2-negative participants as tested by RT–qPCR on the day of blood sampling. The National Review Board approved the study protocol (*Comissão Nacional de Ética em Pesquisa* #30650420.4.1001.0008), and informed consent was obtained from all participants or patients’ legal representatives. Peripheral blood samples were drawn into acid-citrate-dextrose (ACD) and centrifuged at 200 × *g* for 20 min to obtain platelet-rich plasma (PRP). Briefly, PRP was centrifuged at 500 × *g* for 20 min in the presence of 100 nM prostaglandin E_1_ (PGE_1_) (Cayman Chemicals). The supernatant was discarded, and the platelet pellet was resuspended in 2.5 mL of phosphate-buffered saline containing 2 mM EDTA, 0.5% human serum albumin, and 100 nM PGE1 and incubated with anti-CD45 tetrameric antibody complexes (1:25) for 10 min and with dextran-coated magnetic beads (1:50) for an additional 15 min before purification in a magnet (Human CD45 depletion kit; Stem Cell, Easy Sep Technology). Recovered platelets were resuspended in 25 mL of PSG (PIPES–saline–glucose: 5 mM C_8_H_18_N_2_O_6_S_2_, 145 mM NaCl, 4 mM KCl, 50 mM Na_2_HPO_4_, 1 mM MgCl_2_·6H_2_O, and 5.5 mM glucose) containing 100 nM PGE_1_. The platelet suspension was centrifuged at 500 × *g* for 20 min. The supernatant was discarded, and the pellet was resuspended in medium 199 (Lonza). The platelet preparation purity (>99% CD41+) was confirmed by flow cytometry.

### Shotgun proteomics

#### Sample preparation

Platelets were isolated as previously described [12]. The platelet preparation purity (>99% CD41+) was confirmed by flow cytometry. Samples were suspended in 100 µL of 0.2% (w/v) RapiGest SF^®^ (Waters) in 50 mM NH_4_HCO_3_ for cell lysis. The protein concentration was estimated by absorbance reading at 280 nm (NanoDrop 2000, Thermo Scientific), and 100 µg aliquots were subjected to trypsin digestion [12].

#### Mass spectrometry

nLC-nESI MS/MS analysis was performed in an Ultimate 3000 (Dionex) coupled to a Q-Exactive HF-X mass spectrometer (Thermo). Approximately 1 μg of the tryptic digest was applied to a guard column (2 cm × 100 μm internal diameter × 3 μm particle size Magic C18 AQ, Michrom Bioresources) followed by a fractionation column (25.5 cm PicoFrit^TM^ Self-Pack, New Objective × 75 μm internal diameter × 1.9 μm particle size ReproSil-Pur 120 C18-AQ, DR. MAISCH). Mobile phase A (0.1% v/v formic acid in water) and mobile phase B (0.1% v/v formic acid in acetonitrile) were used in a separation gradient from 2% to 40% B for 158 min, reaching 80% B in 4 min, and isocratic elution was performed under this condition for 2 additional minutes. The spray voltage was 1.9 kV in the nanoelectrospray source, and the capillary temperature was set to 250 °C. The lens voltage was set to 60 V. MS1 spectra were acquired in profile mode (*m/z* 300–1500) with a resolution of 70,000 full-width half maximum (FWHM, *m/z* 200). The AGC was set to 1 × 10^6^ with a maximum injection time (IT) of 250 ms. Up to 12 precursor ions per MS1 spectrum were selected for fragmentation with an HCD and NCE of 35, with an activation time of 50 ms. The isolation window was set to 2*m/z*, and the dynamic exclusion was configured to 60 s. MS2 spectra were acquired at a resolution of 17,500 FWHM; AGC was set to 5 × 10^4^, maximum fill of 50 ms, intensity threshold of 1 × 10^5^ counts. Singly charged and unassigned ions were not subjected to fragmentation. Data were obtained in technical triplicate using Xcalibur software (version 4.2.47).

#### Computational analysis

Raw data files were processed and quantified using PatternLab for Proteomics software [64] as previously described [12]. Peptide sequence matching (PSM) was performed against the protein-centric human database NeXtProt [65] plus the SARS-CoV-2 reference proteome from UniProt [66] under ID UP000464024. For protein–protein interaction (PPI) analysis and enrichment analysis of protein sets, the STRING [67] and METASCAPE [68] databases were used. This analysis was performed and filtered by biological process from Gene Ontology [69] terms, and the obtained results were visualized as a functionally organized network in Cytoscape [70].

### Flow cytometry analysis

Platelets (1–5 × 10^6^) were incubated with FITC-conjugated anti-CD41 (BD Phamingen, CA) (1:20) for 30 min at 37 °C. Isotype-matched antibodies were used to control the nonspecific binding of antibodies. Platelets were distinguished by specific binding of anti-CD41 and characteristic forward and side scattering. A minimum of 10,000 gated events were acquired using a FACScalibur flow cytometer (BD Bioscience, CA). Assessment of mitochondrial function was measured using the probe tetramethylrhodamine ethyl ester (TMRE, Fluka Analytical) (100 nM, 10 min) to label mitochondrial membrane potential (ΔΨm). Active caspase-1, caspase 3/7, and caspase-9 were determined using green FAM-YVAD_FMK, FAM-DEVD-FMK, and FAM-LEDH-FMK fluorescent inhibitors of caspases (FLICA), respectively, which irreversibly bind to activated caspases (Immunochemistry Technologies).

### Western blotting

Freshly isolated platelets were lysed [0.15 M NaCl, 10 mM Tris pH 8.0, 0.1 mM EDTA, 10% (v/v) glycerol and 0.5% (v/v) Triton X-100] in the presence of a protease inhibitor cocktail (Roche, Indianapolis, IN). Platelet proteins (20 μg) were separated by SDS–containing 15% polyacrylamide gel electrophoresis (SDS–PAGE) and transferred to nitrocellulose membranes. We used primary mouse anti-human ISG15 (1:500), rabbit anti-human IFTM-1 (1:1000), mouse anti-human caspase-4 (1:1000), or mouse anti-human β-actin (Sigma Aldrich) (1:20,000) antibodies. membranes were revealed using peroxidase-conjugated secondary antibodies (Vector) (1:10,000).

### Cell culture, SARS-CoV-2 virus, and in vitro cell infection

CALU-3 cells (human lung epithelial adenocarcinoma cell line—ATCC/HTB-55) were cultivated, and African green monkey kidney cells (Vero subtype E6) were cultured as previously described [71]. Fresh human platelets from healthy donors were purified as described above. SARS-CoV-2 was originally isolated from nasopharyngeal swabs of the confirmed case from Rio de Janeiro/Brazil (GenBank accession no. MT710714). The virus was amplified in Vero E6 cells in high-glucose DMEM supplemented with 2% FBS and incubated at 37 °C in 5% CO_2_ for 2–4 days of infection. Virus titers were determined by the tissue culture infectious dose at 50% (TCID50/mL), and the virus stocks were kept in −80 °C freezers. According to WHO guidelines, all virus culture procedures were performed in a biosafety level 3 (BSL3) multiuser facility. SARS-CoV-2 infections were performed at an MOI of 0.01 in all cells for 1 h and maintained after the infection. The plaque-forming assay was performed for virus titration in Vero E6 cells seeded in 96-well plates. Cell monolayers were infected with different dilutions of the supernatant containing the virus for 1 h at 37 °C. The cells were overlaid with high glucose DMEM containing 2% FBS and 2.4% carboxymethylcellulose. After 3 days, the cells were fixed with 10% formaldehyde in PBS for 3 h. The cell monolayers were stained with 0.04% crystal violet in 20% ethanol for 1 h. The viral titer was calculated from the count of plaques formed in the wells corresponding to each dilution and expressed as plaque-forming units per mL (PFU/mL).

### Molecular analysis

#### Viral load quantitation

Total RNA was extracted from platelet samples with the QIAamp Viral RNA kit (Qiagen, Germany). Quantitative RT–qPCR was performed with GoTaq^®^ Probe qPCR and RT–qPCR Systems (Promega) on StepOne™ Real-Time PCR equipment (Thermo Fisher Scientific). Amplification reactions were carried out with 50 µM of each primer, 10 µM of probe, and 5 µL of RNA template, following the protocol recommendations by the Centers for Disease Control and Prevention (CDC) to detect SARS-CoV-2 [26]. The housekeeping gene RNAse P was used as a reference for endogenous cells, and the delta-delta Ct method (i.e., 2^–∆∆Ct^) was used to quantify the relative fold change in viral load in each sample.

#### SARS-CoV-2 sequencing

SARS-CoV-2 complete genomes were recovered from platelet samples, following amplicon-based massively parallel sequencing with the ATOPlex SARS-CoV-2 Full Length Genome Panel v1.0 (kindly donated by MGI Tech Co., Shenzhen, China). Briefly, total RNA was reverse-transcribed to cDNA and submitted to consecutive amplification rounds for enrichment and dual indexing. cDNA libraries were then normalized, pooled, circularized, and digested into single-stranded molecules. Finally, ssCirDNA was converted to DNA nanoballs by rolling circle amplification and submitted to paired-end sequencing (100 nt) on the MGISEQ-2000 platform (aka. DNBSEQ-G400) (MGI Tech Co. Ltd., Shenzhen, China).

#### Computational analysis

Viral genome sequences were uncovered by submitting the raw data to a validated online platform (Genome Detective Virus Tool, version 1.132) that conducts a workflow for quality scoring, filtering out nonviral host contaminants, adaptor trimming, contig assembly, consensus calculation, and species ID assignment [72]. SARS-CoV-2-specific BAM alignments were analyzed with integrated bioinformatics tools in Unipro UGENE v39 [73–75], NCBI Genome Workbench [76], and the web-based Galaxy platform. Coverage graphs were obtained by rendering the alignment data into log-scaled histograms (bin size = 20 bases).

## Supplementary information

Supplemental Table 1

Supplemental Table 2**

Supplemental Table 3

Supplemental Table 4

Supplemental Figure 1

Supplemental Figure 2

Supplemental Figure 3

## Data Availability

The proteomics data have been deposited to the ProteomeXchange Consortium via the PRIDE [77] partner repository with the dataset identifier PXD031251. The SARS-CoV-2 genome consensus data have been deposited in GenBank under the accession numbers listed in Supplemental Table 4. Please direct other inquiries to the corresponding author: monique.trugilho@fiocruz.br.
