# Supplementary material for: Platelet proteome reveals features of cell death, antiviral response and viral replication in covid-19
Source: Cell Death Discov. 2022 Jul 16;8:324. doi: 10.1038/s41420-022-01122-1 (PMC9287722; doi:10.1038/s41420-022-01122-1)
Supplement: Supplementary file 4 — Supplemental Table 4 [file 41420_2022_1122_MOESM4_ESM.docx]

| **Supplemental Table 4 -** NGS data statistics summary of platelet samples.  ^a^ Superscript numbers refer to technical replicates.  ^b^ GenBank accession numbers for consensus genome sequences. | | | | | | | | | | | | | | |
| --- | --- | --- | --- | --- | --- | --- | --- | --- | --- | --- | --- | --- | --- | --- |
|  | |  | | |  |  | |  | |  | |  | |  |
| **Sample ID^a^** | **GenBank^b^** | | **Contigs** | **Reads** | | | **Coverage (%)** | | **Depth of Coverage** | | **NT Identity (%)** | | **AA Identity (%)** | |
| IC01041 | OL984052 | | 3 | 670,497 | | | 99.9 | | 1,974.8 | | 99.9 | | 99.8 | |
| IC01048^1^ | OL984053 | | 2 | 5,009,853 | | | 99.9 | | 14,865.0 | | 99.2 | | 98.8 | |
| IC01048^2^ | OL984054 | | 1 | 4,966,309 | | | 99.8 | | 14,742.7 | | 99.3 | | 98.8 | |
| IC01048^3^ | OL984055 | | 2 | 4,909,184 | | | 99.9 | | 14,559.1 | | 99.3 | | 98.9 | |
| IC01048^4^ | OL984056 | | 1 | 4,870,938 | | | 99.8 | | 14,470.7 | | 99.3 | | 98.9 | |
| IC01049^1^ | OL986402 | | 2 | 4,226,399 | | | 99.9 | | 12,539.6 | | 99.0 | | 98.5 | |
| IC01049^2^ | OL986403 | | 1 | 4,197,928 | | | 99.8 | | 12,455.5 | | 99.0 | | 98.6 | |
| IC01049^3^ | OL986404 | | 2 | 4,147,443 | | | 99.9 | | 12,293.8 | | 98.9 | | 98.6 | |
| IC01049^4^ | OL986405 | | 1 | 4,097,538 | | | 99.8 | | 12,189.3 | | 99.0 | | 98.5 | |
| IC01050^1^ | OL984057 | | 2 | 2,974,084 | | | 99.9 | | 8,824.0 | | 99.3 | | 99.0 | |
| IC01050^2^ | OL984058 | | 1 | 2,947,905 | | | 99.8 | | 8,764.1 | | 99.2 | | 99.0 | |
| IC01050^3^ | OL984059 | | 2 | 2,923,396 | | | 99.9 | | 8,676.8 | | 99.2 | | 99.0 | |
| IC01050^4^ | OL984060 | | 1 | 2,838,390 | | | 99.8 | | 8,465.1 | | 99.3 | | 99.2 | |
| IC01051^1^ | OL984061 | | 1 | 840,953 | | | 99.8 | | 2,475.2 | | 98.5 | | 98.0 | |
| IC01051^2^ | OL984062 | | 1 | 836,854 | | | 99.8 | | 2,464.8 | | 98.5 | | 98.1 | |
| IC01051^3^ | OL984063 | | 1 | 827,395 | | | 99.8 | | 2,433.7 | | 98.4 | | 97.9 | |
| IC01051^4^ | OL984064 | | 1 | 809,003 | | | 99.8 | | 2,382.2 | | 98.5 | | 98.1 | |
