# Supplementary figures and images for: Platelet proteome reveals features of cell death, antiviral response and viral replication in covid-19
Source: Cell Death Discov. 2022 Jul 16;8:324. doi: 10.1038/s41420-022-01122-1 (PMC9287722; doi:10.1038/s41420-022-01122-1)

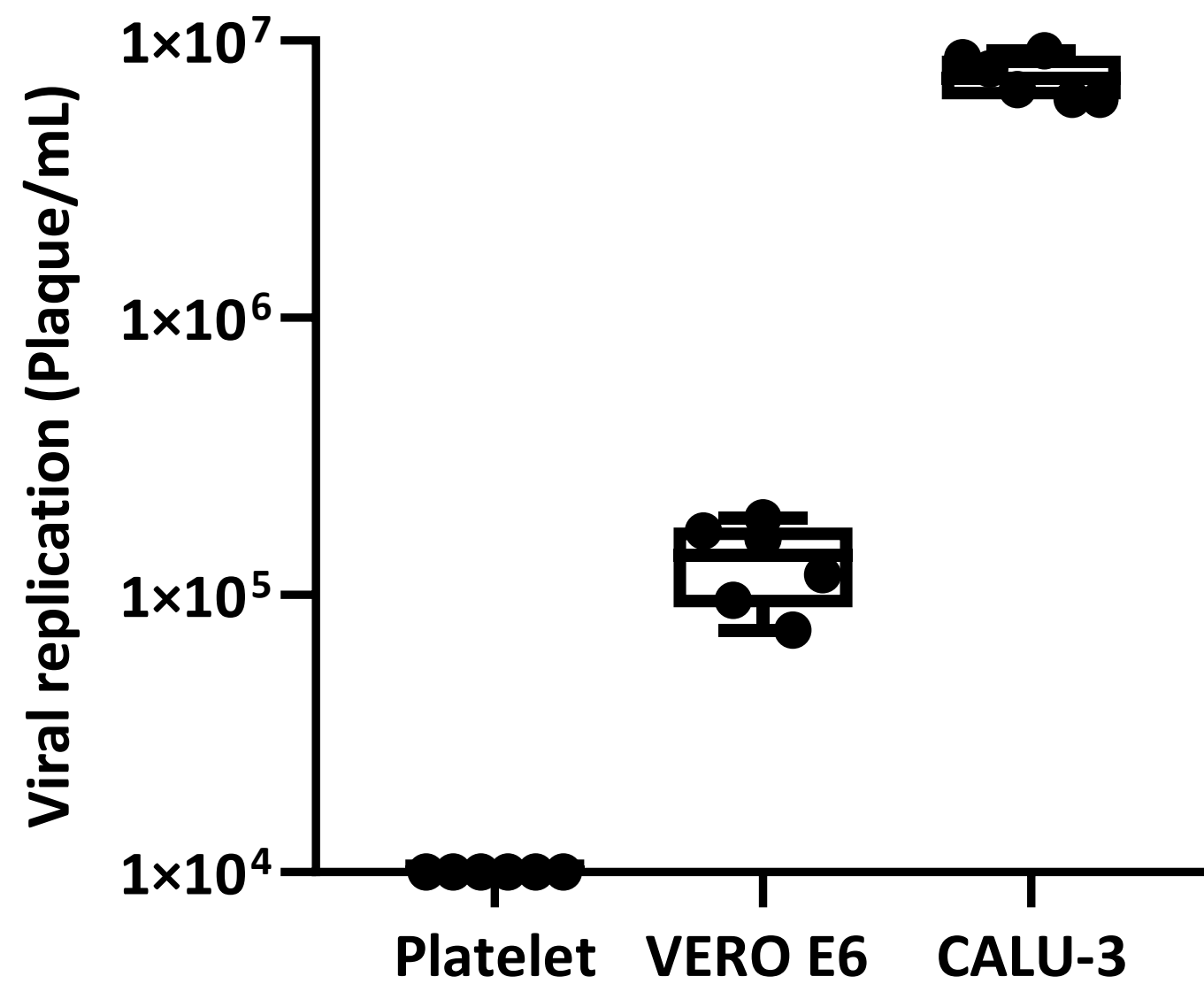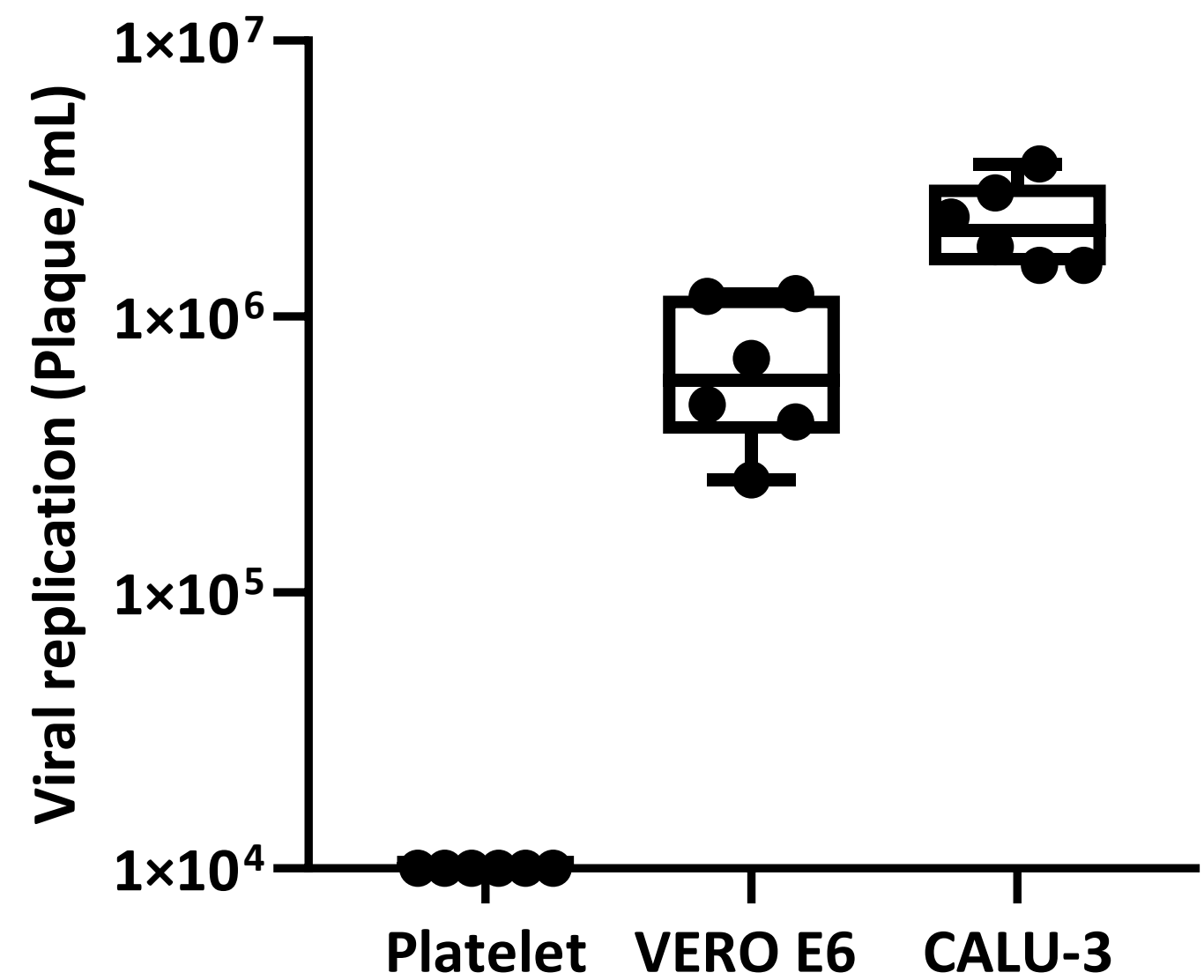

Supplement: Supplementary file 6 — Supplemental Figure 2 [file 41420_2022_1122_MOESM6_ESM.pdf]
