# Supplementary material for: Platelet proteome reveals features of cell death, antiviral response and viral replication in covid-19
Source: Cell Death Discov. 2022 Jul 16;8:324. doi: 10.1038/s41420-022-01122-1 (PMC9287722; doi:10.1038/s41420-022-01122-1)
Supplement: Supplementary file 7 — Supplemental Figure 3 [file 41420_2022_1122_MOESM7_ESM.pdf]

**Supplemental Figure 3:** Original western blot images of caspase 4; IFITM-1; ISG15 and β-actin in platelets isolated from three controls and nine patients with severe COVID-19.

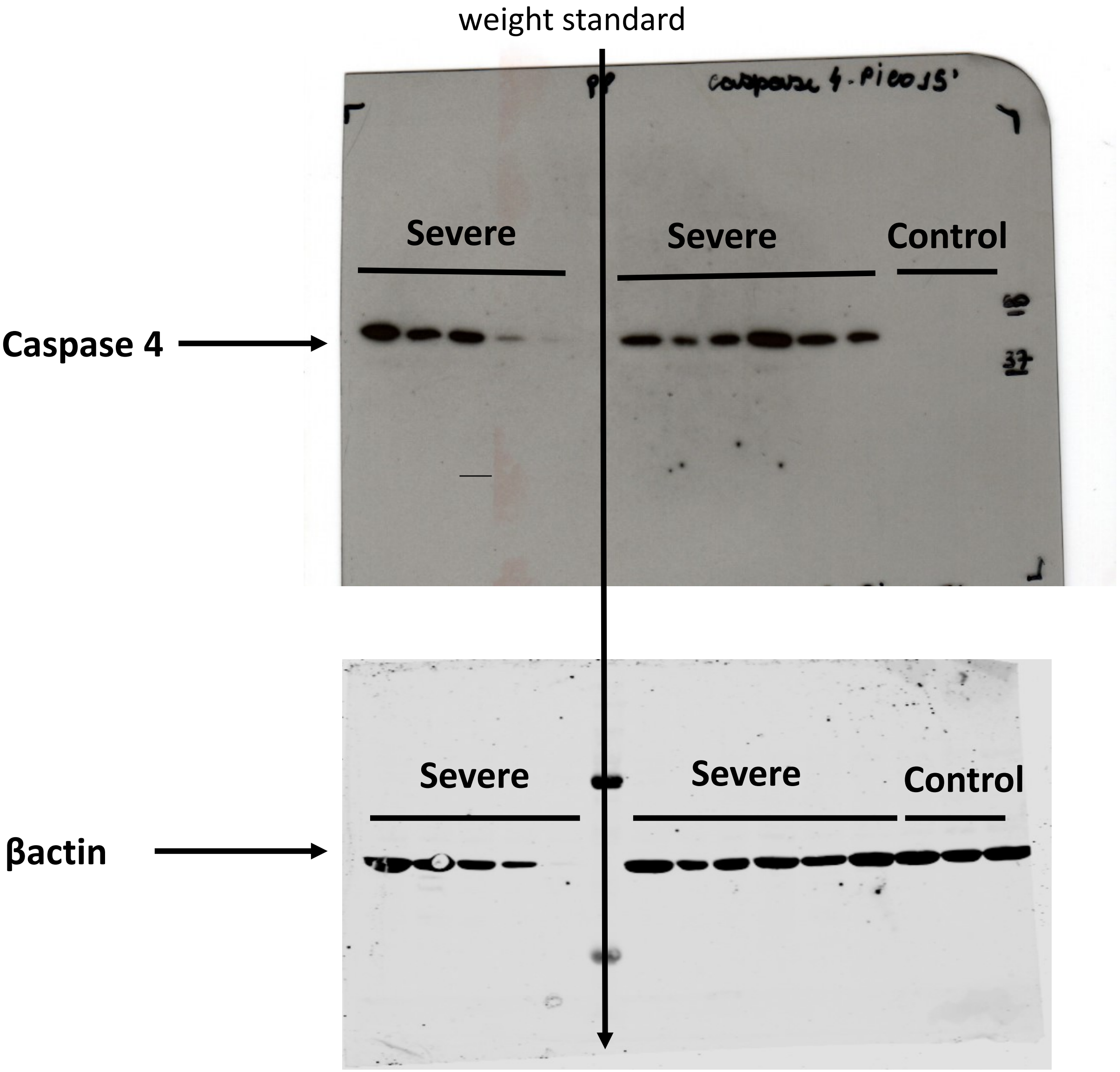

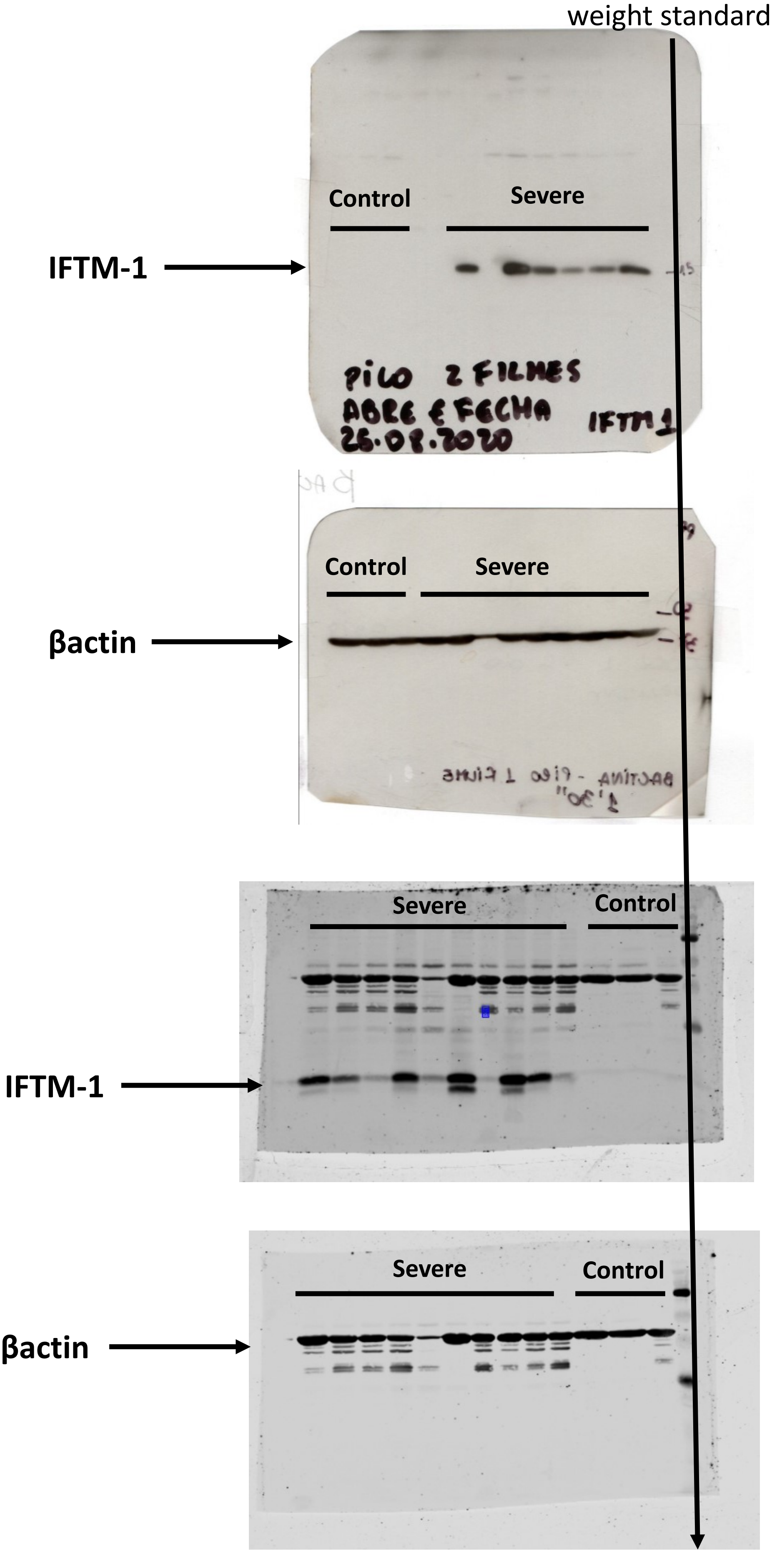
weight standard
Control
Severe
IFTM-1
PILO 2 FILMES
ABRE E FECHA
25.08.2020
IFTM1
Control
Severe
βactin
Severe
Control
IFTM-1
Severe
Control
βactin

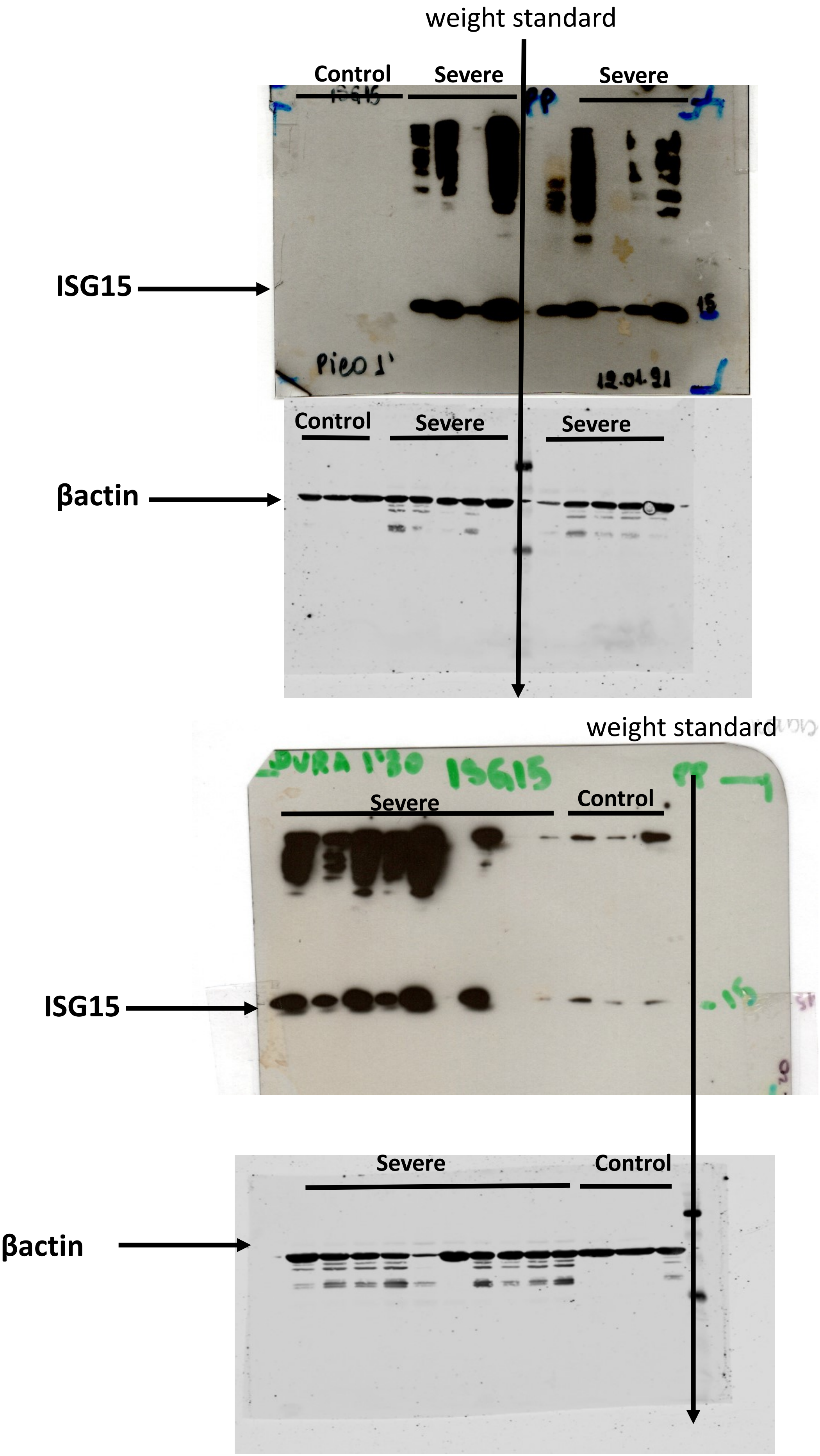
weight standard
Control
Severe
Severe
ISG15
Control
Severe
Severe
βactin
weight standard
Severe
Control
ISG15
Severe
Control
βactin
